# Altered Spontaneous Brain Activity in Primary Open Angle Glaucoma: A Resting-State Functional Magnetic Resonance Imaging Study

**DOI:** 10.1371/journal.pone.0089493

**Published:** 2014-02-24

**Authors:** Yinwei Song, Ketao Mu, Junming Wang, Fuchun Lin, Zhiqi Chen, Xiaoqin Yan, Yonghong Hao, Wenzhen Zhu, Hong Zhang

**Affiliations:** 1 Department of Ophthalmology, Tongji Hospital, Tongji Medical College, Huazhong University of Science and Technology, Wuhan, China; 2 Department of Radiology, Tongji Hospital, Tongji Medical College, Huazhong University of Science and Technology, Wuhan, China; 3 Wuhan Center for Magnetic Resonance, State Key Laboratory of Magnetic Resonance and Atomic and Molecular Physics, Wuhan Institute of Physics and Mathematics, Chinese Academy of Sciences, Wuhan, China; Bascom Palmer Eye Institute, University of Miami School of Medicine;, United States of America

## Abstract

**Background:**

Previous studies demonstrated that primary open angle glaucoma (POAG) is associated with abnormal brain structure; however, little is known about the changes in the local synchronization of spontaneous activity. The main objective of this study was to investigate spontaneous brain activity in patients with POAG using regional homogeneity (ReHo) analysis based on resting state functional magnetic resonance imaging (rs-fMRI).

**Methodology/Principal Findings:**

Thirty-nine POAG patients and forty-one age- and gender- matched healthy controls were finally included in the study. ReHo values were used to evaluate spontaneous brain activity and whole brain voxel-wise analysis of ReHo was carried out to detect differences by region in spontaneous brain activity between groups. Compared to controls, POAG patients showed increased ReHo in the right dorsal anterior cingulated cortex, the bilateral medial frontal gyrus and the right cerebellar anterior lobe, and decreased ReHo in the bilateral calcarine, bilateral precuneus gryus, bilateral pre/postcentral gyrus, left inferior parietal lobule and left cerebellum posterior lobe. A multiple linear regression analysis was performed to explore the relationships between clinical measures and ReHo by region showed significant group differences in the POAG group. Negative correlations were found between age and the ReHo values of the superior frontal gyrus (r = −0.323, p = 0.045), left calcarine (r = −0.357, p = 0.026) and inferior parietal lobule (r = −0.362, p = 0.024). A negative correlation was found between the ReHo values of the left precuneus and the cumulative mean defect (r = −0.400, p = 0.012).

**Conclusions:**

POAG was associated with abnormal brain spontaneous activity in some brain regions and such changed regional activity may be associated with clinical parameters. Spontaneous brain activity may play a role in POAG initiation and progression.

## Introduction

Primary open angle glaucoma (POAG), a neurodegenerative optic disease, is the leading cause of irreversible blindness in the world. Clinical features include optic nerve rim loss, retinal nerve fiber damage and visual field defect. The risk of glaucomatous optic neuropathy increases with multiple risk factors, including elevated intraocular pressure (IOP), age greater than 60 years, a family history of glaucoma and African race. However, a fundamental understanding of the pathology and mechanism of POAG is important because, thus far, no comprehensive theory has been developed to explain all clinical presentations. Previous studies agree with that not only does POAG injure the optic nerve but cross-synaptic degeneration also extends into the lateral geniculate body, as well as to the tertiary neuron in the visual cortex [1]–[7].

Recently, advanced magnetic resonance imaging (MRI) technology permits visualization of minor changes in the whole brain in vivo. We applied both techniques of proton density sequence and gray matter sequence to measure the height and volume of the lateral geniculate nucleus in POAG. Both sequences revealed that the maximum height and volume of the lateral geniculate nucleus in POAG patients were significantly reduced compared with healthy volunteers and correlated with cumulative clinical glaucoma stage [8], [9]. The diffusion tensor imaging technique can detect myelin and axonal damage, which represent nerve fiber remodeling. Previous studies have shown that the diffusion tensor imaging parameters of fractional anisotropy, mean diffusivity and radial diffusivity are altered in the optic nerve, chiasm and optic radiations of glaucoma patients [10]–[13]. Voxel-based morphometry (VBM) studies have shown that neural degeneration extends to the primary visual cortex, as well as to other related cortical areas [11], [14]–[19]. Furthermore, some studies have focused on whole brain changes within and beyond the visual pathway in glaucoma [14], [19]. However, all of the above-mentioned techniques only show neuronal morphological changes in POAG, and there is a lack of direct evidence of changes in brain function. Resting-state functional magnetic resonance imaging (rs-fMRI) can show both spontaneous brain activity and the endogenous/background neurophysiological processes of the human brain. Blood oxygenation level-dependent (BOLD) technique, an imaging technique that detect changes in the concentration of oxygenated and deoxygenated hemoglobin, reflecting neuronal activity. Regional homogeneity (ReHo) analysis based on BOLD is widely used to detect the function of various brain areas. Taking into account the previous studies and the most current MRI techniques, we used BOLD technique to quantify the spontaneous change in neuronal activity in patients with POAG and in matched controls in the resting state.

## Materials and Methods

### Subjects

From October 2010 to January 2013, 42 patients with POAG who were admitted to the Ophthalmology Department at Tongji Hospital, Medical College, Huazhong University of Science and Technology and who met the following criteria were prospectively included: (1) one or both eyes with glaucomatous-type optic disc cupping, or patients with thinning of the inferior and/or superior rim with a cup/disc ratio (CDR) asymmetry >0.2; (2) normal anterior chamber with an open angle in both eyes; (3) matched glaucomatous visual field defects; and (4) right-handedness. The control group was comprised of age- and gender-matched healthy subjects. Exclusion criteria were secondary glaucoma and any other ocular or neurological disorder that could affect the optic visual pathway. Subjects unable to undergo MRI scanning due to metal implantation or those with a history of cigarette consumption, claustrophobia or other psychological disorders were excluded.

Forty-two POAG patients (33 males and 9 females) met the inclusion criteria: a total of 39 formed the POAG group (two patients had head movement >3 mm, and one patient had incomplete data). Of the 42 age- and gender-matched controls selected, 41 formed the control group (one had incomplete data).

The cumulative clinical measurement, such as mean defect (MD), retinal nerve fiber layer thickness (RNFLT), cup to disc ratio (CDR) and intraocular pressure (IOP) was calculated as the sum of the measurements of both eyes in each individual.

Table 1 shows the demographic information and clinical measures for POAG patients and healthy controls. POAG patients were matched with the controls in age (p = 1.00) and gender (p = 0.86) distribution. There were significant differences in the best-corrected visual acuity (p<0.0001), CDR (p<0.0001), MD and RNFLT (p<0.0001) of the bilateral eyes, and the IOP of the left eye (p<0.027), while a trend difference in the IOP of the right eye (p<0.095) was seen between groups.

**Table 1:** Demographic and ocular characteristics (mean±standard deviation) in patients with primary open-angle glaucoma (POAG) and control group.

Issues	Groups		P
	POAG	Normal Control	
Male/Female	32/7	33/8	
Age (years)	34.82±9.90	34.83±9.65	0.997
Best-corrected VA-Right	0.80±0.42	1.12±0.10	<0.001
Best-corrected VA-Left	0.78±0.43	1.14±0.11	<0.001
Refraction (D)-Right	−2.74±2.87	−1.85±2.09	0.116
Refraction (D)-Left	−2.55±2.79	−1.78±1.97	0.156
IOP-Right	17.08±8.71	14.34±2.30	0.056
IOP-Left	17.33±6.47	14.44±2.38	0.011
CDR-Right	0.81±0.17	0.30±0.05	<0.001
CDR-Left	0.77±0.23	0.31±0.06	<0.001
MD-Right	−16.09±9.76	−1.35±0.93	<0.001
MD-Left	−13.64±13.94	−1.53±0.99	<0.001
RNFLT-Right	58.22±21.59	111.28±8.29	<0.001
RNFLT-Left	66.62±26.16	111.27±8.29	<0.001

VA visual acuity; D diopters; IOP intraocular pressure; CDR cup/disc ratio; MD mean defect; RNFLT retinal nerve fiber thickness.

The study followed the tenets of the Declaration of Helsinki and was approved by the Ethics Committee of the Tongji Hospital, Medical College, Huazhong University of Science and Technology Institute, Wuhan, P.R. China. Written informed consent was obtained from subjects after the purposes of the research study were explained. The individuals in this manuscript gave written informed consent to publish these case details.

### Data Acquisition

All subjects were examined by a 3 T magnetic resonance scanner (Signa HDxt, GE Healthcare, Milwaukee, WI) with an 8-channel phased-array head coil. The rs-fMRI data were acquired with the following parameters: repetition time = 2,000 ms; echo time = 30 ms; flip angle = 90°; acquisition matrix = 64×64; field of view = 240×240 mm2; slice thickness = 3 mm with a 1 mm gap. Each brain volume comprised 33 axial slices and each run contained 240 volumes. During the rs-fMRI data acquisition, subjects were instructed to keep their eyes closed, relax their minds but not fall asleep, and remain as motionless as possible.

### Data Processing

Data processing was carried out using SPM8 (http://www.fil.ion.ucl.ac.uk/spm). For each subject, the first 10 volumes were discarded to allow for magnetization equilibration. The remaining volumes were corrected for the acquisition time and realigned to correct for head motion. Subjects with maximum displacement in any direction of more than 3.0 mm or head rotation of more than 3.0°were excluded from this study. Subjects with incomplete data were also excluded. As a result, data of three POAG patients and one control subject were excluded and a total of 80 subjects (39 POAG patients and 41 controls) were analyzed. The realigned images were then spatially normalized to the Montreal Neurological Institute space and resampled to 3 mm isotropic voxels. Finally, the linear trend of the time series was removed and band-pass filtering (0.01–0.08 Hz) was performed to reduce the effects of physiological noise.

ReHo was used to evaluate spontaneous brain activity in POAG patients. ReHo analysis was carried out using the Resting-State fMRI Data Analysis Toolkit (http://restfmri.net/forum/REST). ReHo was defined as the Kendall’s coefficient of concordance (KCC) of the time series of a given voxel with its nearest 26 neighboring voxels. Each individual ReHo map was generated by a voxel-wise manner [20]. Finally, the ReHo maps were smoothed by 6 mm full width at half maximum (FWHM) Gaussian kernel.

### Statistical Analysis

A voxel-wise two-sample t-test with age and gender as covariates was performed to detect group differences in ReHo across the whole brain between POAG patients and healthy controls. The statistical map was set at a combined threshold of p<0.005 for each voxel with a minimum cluster size of 26 voxels (702 mm^3^), resulting in a corrected threshold of p_alpha_<0.05 as determined by Monte Carlo simulation (AlphaSim with the following parameters: single voxel p = 0.005, FWHM = 6 mm, cluster connection radius r = 5 mm, with the Automated Anatomical Labelling template as a mask).

Additionally, although the groups were well matched for age and gender, brain function is still highly dependent on age, a fact that must be taken into account as a confounding variable in any analysis involving brain function. An analysis of stepwise multiple linear regression analysis (ANCOVA) with age, gender, cumulative IOP, cumulative MD, cumulative CDR and cumulative RNFLT as covariates was performed to assess the relationships between clinical variables and spontaneous brain activity in the affected regions showing changed ReHo values in POAG patients. A p value of less than 0.05 was considered statistically significant. Statistical analyses were performed by using the Statistical Product and Service Solutions Statistics, Version 20.0 for Windows (IBM SPSS Statistics-win64).

## Results

**Figure 1:**
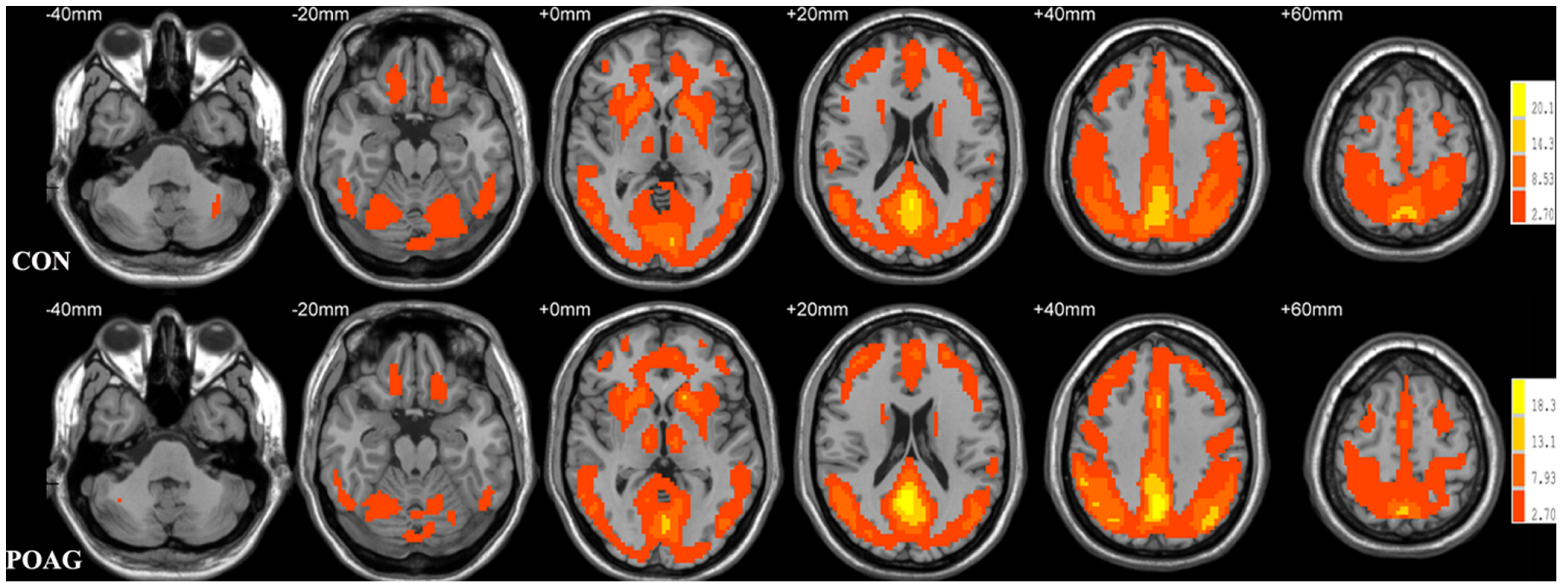
Results from one-sample t-test on ReHo maps for healthy subjects (CON, upper) and POAG patients (lower). Threshold was set to p<0.05 with AlphaSim correction.

The within-group ReHo maps for the healthy controls and POAG patients were shown in Figure 1. For both the two groups, extensive grey matter regions showed significant larger-than-global-mean ReHo values. Those regions include sensorimotor areas, visual areas, auditory areas, prefrontal cortex, temporal cortex, parietal cortex, insula, cerebellum, striatum, thalamus and the default mode network.

**Figure 2:**
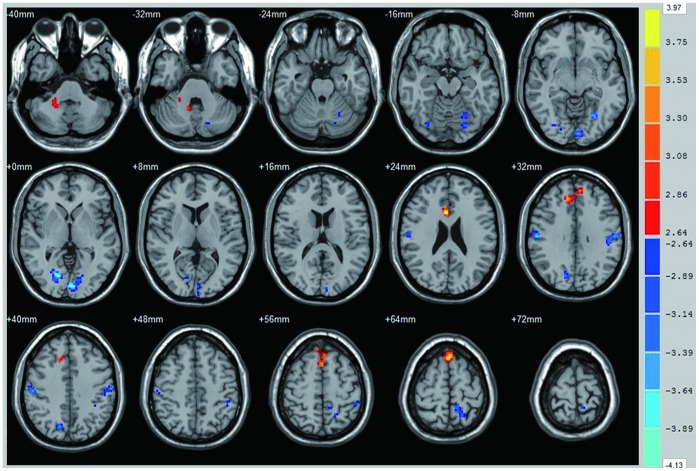
Significant differences of spontaneous brain activity between the POAG group and normal control group (p<0.05 with AlphaSim correction). The right dorsal anterior cingulate, bilateral medial superior frontal gyrus, bilateral medial frontal gyrus and right cerebellum anterior lobe showed increased spontaneous brain activity in POAG group (red). The bilateral calcarine, right lingual gyrus, bilateral precuneus gyrus, left cerebellum posterior lobe, left inferior parietal lobule, bilateral precentral and postcentral gyrus showed decreased spontaneous brain activity in POAG group (blue).

Compared with healthy controls, POAG patients had significantly increased ReHo values in the brain regions of the right dorsal anterior cingulated cortex, the bilateral medial frontal gyrus and the right cerebellar anterior lobe (Figure 2 red and Table 2). Brain areas with significant decreased ReHo values in POAG were located in the bilateral calcarine, bilateral precuneus, bilateral precentral/postcentral gyrus, left inferior parietal lobule and left cerebellar posterior lobe (Figure 2 blue and Table 2).

**Table 2:** Brain regions with significant differences in ReHo between control and POAG group (P(corrected)<0.05, P(uncorrected)<0.005 and cluster voxels>26).

Brain areas	Hemisphere	BA	MNI coordinates (cluster maxima, mm)			Peak T values	Cluster size (voxels)
			X	Y	Z		
**POAG<CON**							
Dorsal Cingulate Cortex	Right	33	6	18	24	3.97	47
Medial Superior Frontal Gyrus	Bilateral		0	27	63	3.71	90
Medial Frontal Gyrus	Bilateral	9	12	36	33	3.45	64
Cerebellum Anterior Lobe	Right		24	−42	−36	3.09	31
**POAG<CON**							
Calcarine	Left	17	−3	−90	0	4.13	132
Calcarine/Lingual Gyrus	Right	18	15	−75	0	4.13	97
Precentral/Postcentral Gyrus	Left	4	−60	−18	36	3.78	57
Precentral/Postcentral Gyrus	Right	3	57	−18	33	3.75	68
Occipital/Cerebellum Posterior Lobe	Left		−27	−63	−9	3.58	87
Precuneus	Left		−12	−45	69	3.55	55
Precuneus	Right		15	−72	39	3.51	39
Inferior Parietal Lobule	Left		−45	−39	48	3.14	27

BA Brodmann area; MNI: Montreal Neurological Institute;

ReHo: regional homogeneity; POAG: primary open-angle glaucoma;

Negative correlations were found between age and the ReHo values of the superior frontal gyrus (r = −0.323, p = 0.04), left inferior parietal lobule(r = −0.362, p = 0.024) and left calcarine (r = −0.357, p = 0.026) in the POAG group but not in the control group.

**Figure 3:**
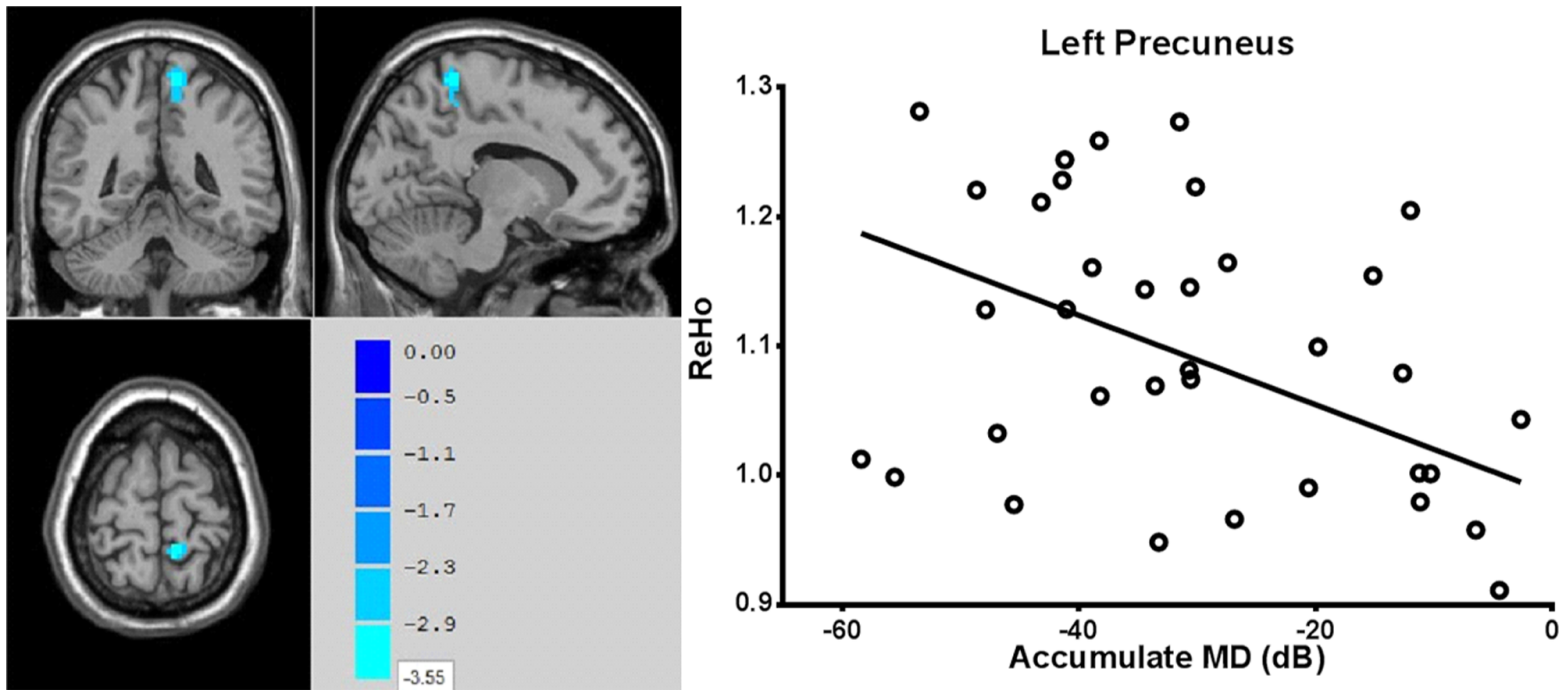
ReHo values of the left precuneus showed negative correlation with cumulative MD values in POAG group (r = −0.400, p = 0.012).

In the POAG group, the ReHo values of the left precuneus were negatively correlated with the cumulative MD (r = −0.400, p = 0.012) (Figure 3).

## Discussion

Glaucoma is well understood at the level of the retina and optic nerve, but remains poorly understood at the level of the whole brain. Various mechanisms such as endoplasmic reticulum stress, dendrite disruption and astrogliosis activation are involved in the plasticity of the neural system, resulting in functional deficits and reorganization of the whole brain network. Many previous MRI studies have shown altered brain structure in POAG patients; however, little has been published regarding where or how spontaneous underlying brain activity differs between POAG patients and healthy subjects. Rs-fMRI technology enables observation of the functional alterations in POAG patients. ReHo, a voxel-wise method of rs-fMRI analysis, was used in the current study to provide rapid mapping of regional activity across the whole brain [20].

Our result is consistent with previous studies which show both morphological atropy and functional deterioration in the visual cortex of POAG patients [19], [21], [22]. However, our study differs Chen’s study in important ways. The previous study found increased gray matter volume in the bilateral middle temporal gyrus and precuneus, and decreased gray matter volume in the superior and inferior frontal gyrus [19]. In our study, on the other hand, neuroactivity was significantly increased in the medial and medial superior frontal gyrus, but not in the middle temporal gyrus. The differences may stem from the fact that that our study included patients with POAG of any stage from 0–5, including bilateral asymmetric optic nerve damage, but Chen’s study included only symmetric terminal stage patients. Another reason may be the different scan sequences and analytical methods used. VBM is a semi-automated technique that detects morphological changes at the whole-brain level, but rs-fMRI uses the BOLD technique to, reflect neuronal activity and changes in functional connections at the whole-brain level. Although in most situations the functional changes agree with the morphological changes, the results are sometimes inconsistent. Therefore, using a combination of both techniques may provide greater insight into the pathological mechanisms of POAG.

In the current study, spontaneous neuronal activity was decreased in the primary visual cortex (BA17) and the secondary visual cortex (BA 18 and 19), caused by apoptosis of retinal ganglion cells. Morphological research indicates that the visual cortex gradually atrophies as POAG progresses [1]. Recently, Duncan revealed decreased cerebral blood flow in the visual cortex of those with POAG [22], suggesting that regional metabolism and blood flow contribute to the phenomenon. Visual information is known to be transmitted through the dorsal and ventral pathways. The former involves the parietal gyrus and responds to spatial information and motional orientation; the latter pathway extends into the temporal lobe and responds to color and shapes [23]. Previous studies found that the occipital cortex predominantly, but also the temporal-parietal regions constitute an intrinsic connectivity network for visual processing [24], [25]. In a recent investigation using rs-fMRI, Dai et al. reported that in POAG patients, voxel-wise analysis showed decreased functional connectivity decreased between the primary visual cortex and the right inferior temporal, left fusiform, left middle occipital, right superior occipital, left postcentral, right precentral gyri and the anterior lobe of the left cerebellum [26]. Obviously, the most affected area of functional connectivity was located in the ventral pathway in Dai’s study; the dorsal pathway changed little. However, clinical features indicate injury to both visual pathways [27]. This contradiction arises from the method by which functional connectivity is measured. Since connectivity represents a correlation of two spatially distinct regions, it is modulated by cerebral blood flow from both regions [28]. Our measuring method has the advantage of clarifying the spontaneous brain activity and cerebral blood flow of each independent region. We detected decreased regional activity in the precuneus and inferior part of the parietal lobe, but no change in regional activity in the temporal lobe. The precuneus is located in the superior region and connects with visual areas in the cuneus and with the primary visual cortex. Functional imaging studies have found that the precuneus and the inferior parietal lobe play an important role in a wide spectrum of highly integrated tasks, such as visuospatial imagery, episodic memory retrieval, self-reflection, and aspects of consciousness [29]–[31]. Therefore, decreased regional activity in the precuneus and the inferior parietal lobe could reflect impaired visual function in the POAG group. The potential mechanism for the decrease in spontaneous activity in the precentral/postcentral gyrus is unknown, but a previous study indicated that the precentral/postcentral gyrus is associated with primary visual areas in the resting state [32]. Although little is known on the mechanism of the decreased activity in the observed above regions, but the neurons accept similar stimulate may have similar structures, and this could partly explain why decreased activity is occurring in some but not the whole brain [33].

The regions of hyperactivity in the current study include the right anterior cingulate cortex (ACC) and the bilateral medial frontal gyrus. The dorsal region of the ACC is the cognitive subdivision of the ACC. It is connected with the prefrontal cortex, the parietal cortex, the motor system and the frontal eye fields, and modulates the frontal eye field, visual attention particularly during the tracking of visible targets [34]; it is also involved in the categorical recognition of visual objects [35]. Clinically, when the right dorsal ACC is invaded by a mass, such as a tumor, visual function deficits occur; once the tumor is removed, visual attention and visual memory improves [36]. The frontal lobe helps in the optic localization, coordinating multiple retinal fields and determining the fixation point. It processes the coordination of spatial information beyond the categorical processing [37], and transmits the visual information to the oculomotor complex [38]. The frontal lobe also processes language tasks [39]. Increased neuronal activity in the superior and medial frontal gyrus is due to cerebral plastic change, including neurodegeneration and structural reconstruction. In the current study, we also found that regional activity was increased in the left cerebellar posterior lobe, but decreased in the right cerebellar anterior lobe. This results partly confirms the notion that different cerebellar subregions play different roles in different intrinsic connectivity networks [40].

In the POAG group but not the control group, age was negatively correlate with the ReHo values of the superior frontal gyrus, the left inferior parietal lobule and the left calcarine. The results of our study suggests that POAG may aggravate the degeneration of central nerve system that occurs with age, and furthermore, that changes in distant neuronal populations may aggravate of existing central nervous system disease [41].

Interestingly, a negative correlation was also found between the ReHo values of the left precuneus and the cumulative MD in POAG group(r = −0.400, p = 0.012). A previous study reported that the precuneus plays a role in peripheral vision [42]. It is difficult to determine why there is a negative correlation between the spontaneous activity in this region and cumulative MD, because this region had decreased activity. However, a very recent study indicated that, early in POAG, the brain goes through a temporary compensated stage in which most brain structures become larger, before they develop structural atrophy [43]. This brain change is a result of the defense mechanism of suppressing neuronal activity at the early stage of the disease, accompanied by a compensatory mechanism of increasing neuronal activity and increasing dendritic arborization and axonal tracts [44].

Some limitations in our research should be mentioned. Firstly, the number of subjects included in the study was relatively small for obtaining subgroup differences within the POAG group. However, this emerging tool showed statistically significant differences between the POAG group and the control group. The underlying mechanism of these intriguing results, once elucidated, promises to provide further insight into the neurobiology and functional consequences of POAG. Secondly, an unresolved issue is the number of independent clusters needed to neither overestimate the regional activity nor over-split the main networks [45]. Thirdly, the current research is just a first step toward understanding the abnormal condition in the resting state in POAG patients, which may play an important role in neuronal functional reconstruction and POAG development. The next step is to determine the different connections of the resting state networks. Fourth, although some previous researches indicated that some areas were rhythm-related and IOP-related, we found no correlation between IOP and spontaneous brain activity. The most important reason may be most subjects were received IOP-lowing therapy, so that there was no significant difference in IOP between two groups. And it is still unclear why some area differences are unilateral.

In conclusion, rs-fMRI reveals, abnormal regional spontaneous activity involved in brain changes associated with POAG. Our study, showing a negative correlation between the spontaneous activity of the precuneus and clinical severity, enhances our understanding of the retinotopy in the brain. Future studies are needed to determine the complete structural and functional changes that occur from the early to the end stage of POAG.
